# Random access with adaptive packet aggregation in LTE/LTE-A

**DOI:** 10.1186/s13638-016-0531-6

**Published:** 2016-02-03

**Authors:** Kaijie Zhou, Navid Nikaein

**Affiliations:** Huawei Technologies Co., Ltd., Shanghai, China; Mobile Communication Department, Eurecom, Campus Sophia-Tech, Biot Sophia-Antipolis, France

**Keywords:** LTE/LTE-A, Random access procedure, Packet aggregation, Semi-Markov model, Traffic source

## Abstract

While random access presents a promising solution for efficient uplink channel access, the preamble collision rate can significantly increase when massive number of devices simultaneously access the channel. To address this issue and improve the reliability of the random access, an adaptive packet aggregation method is proposed. With the proposed method, a device does not trigger a random access for every single packet. Instead, it starts a random access when the number of aggregated packets reaches a given threshold. This method reduces the packet collision rate at the expense of an extra latency, which is used to accumulate multiple packets into a single transmission unit. Therefore, the tradeoff between packet loss rate and channel access latency has to be carefully selected. We use semi-Markov model to derive the packet loss rate and channel access latency as functions of packet aggregation number. Hence, the optimal amount of aggregated packets can be found, which keeps the loss rate below the desired value while minimizing the access latency. We also apply for the idea of packet aggregation for power saving, where a device aggregates as many packets as possible until the latency constraint is reached. Simulations are carried out to evaluate our methods. We find that the packet loss rate and/or power consumption are significantly reduced with the proposed method.

## Introduction

Reliable and low-latency protocols and access methods are becoming crucial to lower the bit error rate and energy consumption and to improve the spectral efficiency in end devices. This is important for the emerging application scenarios found in the massive internet of things (IoT), ultra-reliable, and/or real-time communications [1]. However, the majority of wireless systems, including long-term evolution(LTE)/LTE-advanced (A), are designed to support a continuous flow of information, at least in terms of the timescales needed to send several IP packets, such that the induced signaling overhead is manageable. While these systems are intended mostly for downlink-dominant traffic, emerging application scenarios such as machine-type communication (MTC), online interactive gaming, social networking, and instant messaging are of generally uplink-dominant periodic and event-driven traffics with small and low duty cycle packets [2, 3]. In particular, analysis of the emerging MTC application scenarios has revealed that in majority of cases, the following requirements have to be met, namely:

1. Fast reaction time when massive number of realtime, potentially coordinated, event occurs.
2. Efficient energy consumption for battery-powered devices.
3. Reliable transmission for sporadic traffics especially when there are massive number of coordinated transmissions.

The abovementioned traffic characteristics and requirements impose great challenges on the current and future cellular systems. The legacy LTE/LTE-A offers three access methods, namely random access, (semi-)persistent scheduling, and dynamic scheduling. Random access is the primary uplink channel access that plays a crucial role in supporting efficient and reliable communication, and generally, it can be used to (i) transport the scheduling request (SR) using common random access resources, (ii) transit from the Radio Resource Control (RRC) idle state to the connected state, and (iii) to become uplink synchronized for RRC connected devices. However, the preamble collision rate increases significantly when the number of active terminals increases. In LTE/LTE-A, the maximum available number of preamble in one subframe is 64. Assuming the device number is 1000 and transmission probability for each device is 0.03, the preamble collision probability becomes 0.9997. Moreover, because the traffic patten is often a mix of periodic, event-driven, and burst packets with certain unpredictability, the (semi-)persistent scheduling may not present an efficient solution. Furthermore, dynamic scheduling induces a large signaling overhead and has a limited capacity for the total number of simultaneous SR within one subframe. For example, in LTE/LTE-A, the maximum capacity of SR in one subframe is 36. Therefore, it requires an access period equals to 1000/36 ∗ 1 ms = 28 ms to allocate all SR of all 1000 devices. These limitations call for an optimization in random access channel in view of sporadic, unpredictable, coordinated, and/or delay-bounded traffic sources so as to reduce the collision rate and improve its reliability [4].

Several works have been proposed to improve the performance of random access, with a particular attention to machine-to-machine communication (small low duty cycle packets). Reference [5] investigates a resource allocation scheme for spatial multi-group random access to reduce packet collision in a single cell and interference among multiple cells. Authors in [6] proposes a collision resolution method for random access based on the fixed timing advance information for fixed-location devices in LTE/LTE-A. Authors in [7] introduce a code-expanded method for random access in LTE/LTE-A, where the amount of available contention access resources is expanded to reduce the collision rate. Reference [8] investigates a cooperative random access class barring scheme for global stabilization and access load sharing. In the proposed method, each groups are assigned with specific access class barring to differentiate access priorities. Authors in [9] present a prioritized random access scheme to provide quality of service (QoS) for different classes, where different access priorities are achieved through different backoff procedures. Reference [10] points out some possible directions for controlling the overhead of random access, namely access class barring schemes, separate random access channel (RACH) resource for machine-type device, dynamic allocation of RACH resource, specific backoff scheme for the machine-type devices, slotted access, and pull-based scheme. Splitting the random access preambles into two (non-)overlapping sets, one for human type communication and the other for the machine type communication are proposed by 3GPP [11] as a mean to control the collision rate.

In this paper, we propose an adaptive packet aggregation method for random access applied to LTE/LTE-A, as an extension to our previous work [12], to radically reduce the collision rate and/or power consumption as the number of devices and traffic load increase. The novel contributions of this paper are

- improve the accuracy of the modeling methodology
- validate the analytical model through simulation
- apply the packet aggregation method to save the power under the latency constraint
- support of multiple traffic sources with different QoS requirements

With the proposed method, a device^1^ does not start a random access for every arrived packet (e.g., RRC connection request or scheduling request). Instead, it triggers a random access when the number of packets in a device’s buffer reaches a certain threshold and then aggregate the buffered packets together into a single transmission unit in order to reduce random access attempts. In the above example, with 64 preamble, 1000 devices, and 0.03 transmission probability, aggregating 5 packets leads to a collision probability of 0.21 instead of 0.9997, which is significantly lower.

However, this reduction in preamble collision rate is obtained at the expense of an extra waiting time which is used to accumulate the data packets. It can be seen that the more collision rate is reduced, the more latency is increased, which may not be desirable for delay-sensitive applications. Thus, we apply a semi-Markov process [13] to model the random access in LTE/LTE-A and subsequently to derive the packet loss rate and channel access latency as functions of the number of aggregated packets. With the derived results, the optimal number of aggregated packets to guarantee the desired packet loss rate while minimizing the latency can be selected. We also apply the idea of packet aggregation to multiple traffic sources with different QoS requirements, namely realtime and non-realtime traffics (also called delay-tolerant and delay-critical). In such a case, different aggregation policies can be applied to different traffic sources to achieve an overall trade-off between loss rate and latency. For example, a policy for non-realtime traffic with elastic delay requirement may induce higher level of aggregation, while another policy for realtime traffic with strict delay requirement may render lower level of packet aggregation.

Another benefit that can be exploited from the packet aggregation is the power saving. With packet aggregation, the power consumed by random access is greatly reduced since only one random access is performed for the transmission of multiple packets. It has been shown that crossing the protocol stack significantly contribute to the total power consumption [14, 15], suggesting that the packet aggregation function should be performed closer to the traffic source so that the energy price is only paid for a signal packet.

The remainder of this paper is organized as follows. In Section 2, we highlight the random access mechanism in LTE/LTE-A. The proposed packet aggregation method is presented in Section 3. Model validation and performance results are provided in Section 4. Discussions and usage of the packet aggregation for random access applied to LTE/LTE-A are explained in Section 5. Finally, Section 6 provides concluding remarks.

## Random access mechanism of LTE/LTE-A

**Fig. 1:**
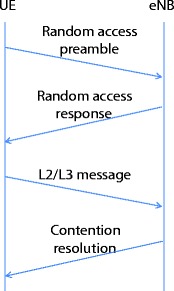
Contention-based random access in LTE/LTE-A

The random access mechanism is specified in [18] and can either be contention-free or contention-based random access. The latter is mainly used for handover and new downlink data transmission for non-uplink-synchronized terminals. The contention-based procedure consists of four steps to be completed as shown in Fig. 1, namely random access preamble transmission (UL), random access response (DL), L2/L3 message (UL), and contention resolution (DL). The following subsections provide further details for each step of the contention-based procedure.

### Random access preamble transmission

A UE randomly selects one of the 64−*N*_*c*_ available random access preambles, where *N*_*c*_ is the number of preambles reserved for contention-free random access. To inform eNB about the packet size of L2/L3 message, the preambles used for contention-based access are further divided into two subgroups based on the eNB allocation: random access preamble subgroups A and B. A UE, whose L2/L3 message size is larger than the parameter *messageSize for group A*, selects a preamble from subgroup B. Otherwise, it uses preambles from subgroup A [18].

### Random access response

After sending the random access preamble, a UE decodes the physical dedicated control channel (PDCCH) within the random access response window to receive the random access response (RAR) message.

The RAR message includes the identity of the detected preamble (random access preamble identifier), uplink channel synchronization information, resource allocation information for the subsequent L2/L3 message transmission, backoff indicator, and the temporary C-RNTI [19]. The backoff indicator is uniformly selected over a period by eNB and is used to instruct UEs to backoff certain time before starting the next random access.

A UE identifies its RAR through the random access preamble identifier that corresponds to the random access preamble transmitted in the first step. Therefore, UEs sent the same preamble in the first step that receives the same RAR message in this stage. If a UE does not receive any RAR after the random access response window, it restarts a new preamble transmission.

### L2/L3 message transmission

In this step, UEs send the L2/L3 message for this random access procedure. Note that a SR will be mainly transmitted to request resources for the uplink data transmission. Multiple L2/L3 messages are sent on the same resource if the same preamble is selected by multiple UEs in the first step. To help an eNB identify collisions, a unique UE identity should be transmitted along with the L2/L3 message.

### Contention resolution reception

eNB acknowledges the successfully decoded L2/L3 message through contention resolution message. The contention resolution message contains identities for UEs whose packets are successfully decoded. Therefore, if a UE detects its own identity, it can infer that the previous L2/L3 message delivery is successful. Otherwise, a new random access procedure is triggered.

## Adaptive packet aggregation method for random access

We introduce an adaptive packet aggregation method for random access in LTE/LTE-A to radically reduce the preamble collision rate and/or power consumption, in particular when the number of contending terminals becomes large. Using the method, a UE does not send a preamble for every signal packet, but rather for multiple aggregated packets. This is achieved at the expense of longer waiting time before the next channel access. Semi-Markov model is applied to analyze the random access procedure in LTE/LTE-A with packet aggregation and to derive the packet loss rate and channel access latency as a function of number of aggregated packets. Hence, the optimal number of aggregated packets, which minimizes the channel access latency and complies with packet loss requirement, can be found.

In the following subsections, two cases are considered for the allocation of random access preamble: single traffic and multiple traffic sources [11]. In the latter case, the assumption is that the available RA preambles are divided into two or more disjoint subsets, and each subset is dedicated to a single machine-type or human-type traffic source. In the former case, the available RA preambles are shared among multiple traffic sources.

### Single traffic source

To analyze, we make use of semi-Markov model (SMM) to accurately capture the random access procedure in LTE/LTE-A [13]. Note that the standard slotted Aloha random access model cannot be applied here as in LTE/LTE-A (i) the number of retransmission is finite, (ii) backlogged and newly generated packets can be transmitted after one random access procedure, and (iii) when a preamble is transmitted, a UE has to wait for certain time to receive the random access response before proceeding with the procedure. Also, the discrete-time Markov chain model proposed to model the distributed coordinated functions (DCF) in IEEE 802.11 is not applicable to LTE/LTE-A [20] as the assumption of saturated traffic cannot be held. Using SMM, each state has its own sojourn time (a.k.a. holding time), where a state is kept for a certain amount of time. Thus, non-saturated traffic can be modeled as an idle state where a terminal dwells until a packet arrives. This calls for a new state to represent the packet generation procedure, whose sojourn time can be calculated as $\frac {1}{\lambda }$, where *λ* is the packet arrival rate. Hence, by the use of SMM, the Bianchi’s model can be extended to analyze the scenario where the traffic is not saturated [20]. The applied method to handle the idle state is similar to those used in [21–26], where the idle state represents the absence of packets.

The proposed SMM, shown in Fig. 2, holds the following assumptions:

1. Packet collides with constant and independent probability, which is similar to the assumption in [20]. Note that this assumption holds when the backoff window and number of UEs are large.

**Fig. 2:**
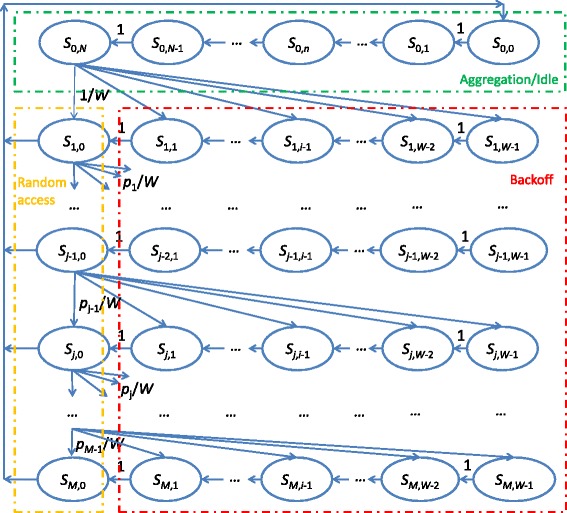
Semi-Markov process model for random access in LTE/LTE-A with packet aggregation
2. All buffered packets can be sent to eNB after one random access procedure. Note that during the random access, new packets may be generated causing the aggregated packet to become larger that the transport block size (i.e., the aggregated packet cannot be transmitted by a single transmission). In such a case, a UE will signal a (padding) buffer status report (BSR) along with the data packet to allow an eNB to adapt the uplink resource allocation accordingly. With this assumption, there is no backlogged packets in the idle state. Therefore, due to this memory-less characteristic, the waiting time for the first packet remains the same $\frac {1}{\lambda }$.
3. Packet arrival is Poisson distributed.
4. Random access opportunity is available in every subframe. Note that this is one of the available 3GPP configuration, namely index 14 [27] and is applicable to massive channel access scenarios [28].
5. Probability *τ* that a station will attempt transmission in one subframe is constant across all subframes [20].

The model includes three types of states, namely aggregation, backoff, and random access, which are described below (see Fig. 2):

- *Aggregation (idle) state*. *S*_0, *n*_,*n*∈ [ 0,*N*] means the random access is not started and there are *n* packets in the UE’s buffer where *N* is the packet aggregation threshold.
- *Backoff state*. *S*_*j*, *i*_,*j*∈ [ 1,*M*],*i*∈ [ 1,*W*−1] means the UE is performing the backoff with a counter size of *i* for the *j*th random access, where *M* is the random access limit and *W* is the maximum backoff counter size.
- *Random access state*. *S*_*j*, 0_,*j*∈ [ 1,*M*] means that the UE is performing the *j*th random access.

The transitions between the states are performed as following:

1. When the UE is at state *S*_0, *n*_,*n*∈ [ 0,*N*−1], for each arrived packet, it transfers to state *S*_0, *n*+1_.
2. When the UE is at state *S*_0, *N*_, it selects a random number *i* which is uniformly distributed over [ 0,*W*−1] and then transfers to state *S*_1, *i*_.
3. When the UE is at state *S*_*j*, *i*_,*j*∈ [ 1,*M*],*i*∈ [ 1,*W*−1], it decreases its backoff counter by 1 after one subframe and transfers to state *S*_*j*, *i*−1_.
4. When the UE is at state *S*_*j*, 0_,*j*∈ [ 1,*M*−1], it starts a random access. If the UE is allocated with some dedicated resource after the random access (the random access is successful), it sends the aggregated packet and transfers to state *S*_0, 0_. Otherwise, it increases the transmission counter by 1 and transfers to state *S*_*j*+1, *i*_, where *i* is uniformly distributed over [ 0,*M*−1].
5. When the UE is at state *S*_*M*, 0_, it performs the random access. If the random access is successful, it sends the aggregated packet on the allocated resource and transfers to state *S*_0,0_. Otherwise, it drops the aggregated packet and transfers to state *S*_0, 0_.

Denoting *p*_*j*_,*j*∈ [ 1,*M*−1], as the unsuccessful probability for the *j*th preamble transmission, the state transition probability from *S*_*j*, 0_,*j*∈ [ 1,*M*−1] to *S*_*j*+1,*i*_,*i*∈ [ 0,*W*−1], is *p*_*j*_/*W*.

An unsuccessful random access can be caused by *wireless channel error* or *collision*; therefore, we have

(1) $$ p_{j}=p_{c}+p^{\prime}_{j}-p^{\prime}_{j}\,p_{c},  $$

where *p*_*c*_ is the preamble collision probability and $p^{\prime }_{j}$ is the error probability caused by wireless channel for the *j*th preamble transmission.

Denoting *π*_*j*, *i*_ as the stationary probability of state *S*_*j*, *i*_, we have

(2) $$ \left\{ \begin{aligned} \overset{} \pi_{0,\,n}& =\pi_{0,\,0}, n\in\,[\!1,N] \\ \pi_{1,\,i}&=\pi_{0,\,N} 1/W+\pi_{1,\,i+1}, i\in\,[\!0,W-2] \\ \pi_{j,\,i}&=\pi_{j-1,\,0} p_{j-1}/W+\pi_{j,\,i+1}, j \in\,[\!2,M], i\in\,[\!0,W-2] \\ \pi_{1,\,W-1}&=\pi_{0,\,N} 1/W \\ \pi_{j,\,W-1}&=\pi_{j-1,\,0} p_{j-1}/W, j \in\,[\!2,M]. \\ \end{aligned}\right.  $$

With the first, second, and fourth equations in equation system (2), we have

(3) $$ \pi_{1,\,0}=\pi_{0,\,N},  $$

(4) $$ \pi_{1,\,i}=\frac{W-i}{W} \pi_{0,\,N}=\frac{W-i}{W} \pi_{1,\,0}, i\in\,[\!1,W-1].  $$

By the use of the third and fifth equations in equation system (2), we get

(5) $$ \pi_{j,\,0}=p_{j-1} \pi_{j-1,\,0}, j \in\,[\!2,M]  $$

(6) $$ \begin{aligned} \pi_{j,\,i}=\frac{W-i}{W}p_{j-1} \pi_{j-1,\,0}=\frac{W-i}{W} \pi_{j,\,0}, j \in\,[\!2,M], i\in\,[\!1,W-1]. \end{aligned}  $$

As the sum of all state stationary probabilities is one, we have

(7) $$\begin{array}{*{20}l} 1&=\sum_{n=0}^{N}\pi_{0,\,n}+\sum_{j=1}^{M}\sum_{i=0}^{W-1}\pi_{j,\,i} \\  &=\pi_{0,\,0}(N+1)+ \sum_{j=1}^{M}\pi_{j,\,0}\sum_{i=0}^{W-1}\frac{W-i}{W} \\  &=\pi_{0,\,0}(N+1)+\sum_{j=1}^{M}\pi_{j,\,0}\frac{W+1}{2} \\  &=\pi_{0,\,0}(N+1)+\frac{W+1}{2}\sum_{j=1}^{M}{\prod_{i=0}^{j-1}p_{i} \pi_{0,\,N}} \\  &=\pi_{0,\,0}\left[\!(N+1)+\frac{W+1}{2}\sum_{j=1}^{M}\prod_{i=0}^{j-1}p_{i}\right], \end{array} $$

where *p*_0_=1. Therefore the stationary probability *π*_0, 0_ can be obtained as

(8) $$ \pi_{0,\,0}=1/\left[N+1+\frac{W+1}{2}\sum_{j=1}^{M}{\prod_{i=0}^{j-1}p_{i}}\right],  $$

which is a function of preamble collision probability *p*_*c*_.

With Eqs. (3) and (5), the stationary probability *π*_*j*, 0_,*j*∈ [ 1,*M*], is given by

(9) $$ \pi_{j,\,0}= \prod_{i=0}^{j-1}p_{i} \pi_{0,\,N}=\prod_{i=0}^{j-1}p_{i} \pi_{0,\,0},  $$

which is also a function of *p*_*c*_.

Now let us calculate the state holding time for this semi-Markov process model. It is obvious that the state holding time for *S*_0, *N*_ and *S*_*j*, *i*_,*j*∈ [ 1,*M*],*i*∈ [ 1,*W*−1] is 1 ms. Moreover, the average state holding time for state *S*_0,*n*_,*n*∈ [ 0,*N*−1] is 1/*λ*. The calculation for the state holding time *S*_*j*, 0_,*j*∈ [ 1,*M*], is less obvious and described in detail below.

Four different events may happen during a random access, each of which has a different state holding time as explained below.

1. The preamble is delivered without collision but with the wireless channel error. The probability for this case is $p_{j}^{\prime }(1-p_{c})$, where *p*_*c*_ is the collision probability and $p_{j}^{\prime }$ is the error probability for the *j*th preamble transmission without collision. If the transmitted preamble is not correctly received by eNB, then no random access response (RAR) will be sent backed to the UE. Therefore, the UE re-starts a random access when the random access response window ends. The state holding time for this event is denoted as *T*_*E*_ (see Table 1).

**Table 1:** Symbols used for single traffic source

Notation	Definition
*S*_*j*, *i*_	State in Semi-Markov process
*N*	Packet aggregation threshold
*λ*	Packet arrival rate
*p*_*j*_	Unsuccessful probability for the *j*th preamble transmission
*M*	Transmission limit for random access
*W*	Backoff window size
$p_{j}^{\prime }$	Error probability caused by wireless channel for the *j*th preamble transmission
*p*_*c*_	Preamble collision probability
*π*_*j*, *i*_	Stationary probability for state *S*_*j*, *i*_
*p*_*E*, *j*_	Probability that a preamble collision cannot be detected by eNB for the *j*th random access
*E*_*i*, *j*_	Probability that a preamble cannot be decoded by eNB when it is sent by *i*+1 UEs for the *j*th random access
*τ*	Probability that as UE is sending a preamble in one subframe
*N*_*C*_	Amount of preamble allocated for contention based random access
*N*_*M*_	Amount of devices in the cell
*T*_*E*_	Duration that starts when the UE sends a preamble and ends at the end of the random access response window
*T*_*C*_	Duration that starts when the UE sends a preamble and ends when the contention resolution timer expires
*T*_*S*_	Duration that starts when the UE sends a preamble and ends when a UE receives the contention resolution message from eNB
*h*_*j*_	Average state holding time for state *S*_*j*, 0_
*Q*_*j*_	Proportion of time that a UE is at state *S*_*j*, 0_
*T*	Average state holding time for all states
$T_{j}^{\prime }$	Duration for an unsuccessful random access at the *j*th try
*T*_*j*_	Channel access latency if the aggregated packet is successfully delivered at *j*th try
*d* ^′^	Expected time used to deliver an aggregated packet
*d*	Overall latency for the first aggregated packet
*α*	Packet loss rate limit
*N*_max_	Maximum allowed amount of aggregated packets
*β*	Power saving factor to measure energy saving with packet aggregation
*d*_0_	Time used for one random access with packet aggregation
2. The preamble is transmitted with collision but cannot be decoded by eNB due to wireless channel error. The probability for this case is *p*_*c*_*p*_*E*, *j*_, where *p*_*E*, *j*_ is the probability that a preamble cannot be detected by eNB when collision happens for the *j*th random access. Assuming amount of preamble allocated for contention based random access is *N*_*C*_, and the number of devices/users in the cell is *N*_*M*_, we can calculate:
  (10) $$ \begin{aligned} p_{E,j}=&\sum_{n=1}^{N_M-1}\binom{N_M-1}{n}\tau^{n}(1-\tau)^{N_M-1-n} \\ &\times\sum_{i=1}^{n}\binom{n}{i}\left(\frac{1}{N_C}\right)^{i}\left(1-\frac{1}{N_C}\right)^{n-i}E_{j,\,i+1}, \end{aligned}  $$
  where *E*_*j,i*+1_ is the probability that a preamble cannot be decoded by eNB when it is sent by *i*+1 UEs for the *j*th random access and *τ* is the probability that a UE is sending a preamble in one subframe. In this case, since the transmitted preamble cannot be correctly detected by eNB, the UE does not receive the RAR message from eNB. Therefore, the state holding time is also *T*_*E*_, which is the same as the first case.
3. The preamble is transmitted with collision but can be decoded by eNB. The probability for this case is *p*_*c*_(1−*p*_*E*, *j*_). This case is quite typical in LTE/LTE-A. For example, if the same preamble is sent by two UEs, since the location of these two UEs are different, two peaks for the same preamble may appear at the eNB side. The probability that all these two peaks cannot be detected by eNB is relative low. As a result, multiple UEs send the L2/L3 messages on the same resource. Assuming that none of the collided L2/L3 messages can be correctly decoded by eNB, the corresponding UE will not receive the contention resolution message. Hence, the UE will restart a random access when the contention resolution timer expires. The state holding time for this event is denoted as *T*_*C*_ (see Table 1).
4. The preamble is successfully delivered without collision and wireless channel error. The probability for this case $\left (1-p_{j}^{\prime }\right)(1-p_{c})$. The state holding time for this event is denoted as *T*_*S*_ (see Table 1).

Hence the expected state holding time for state *S*_*j*, 0_,*j*∈ [ 1,*M*], can be obtained as

(11) $$ \begin{aligned} h_{j}=&p_{j}^{\prime}(1-p_{c})T_{E}+p_{c}p_{E,j}T_{E}+p_{c}(1-p_{E,j})T_{C} \\ &+(1-p_{j}^{\prime})(1-p_{c})T_{S}. \end{aligned}  $$

With the above results, the proportion of time that a UE is at random access state *S*_*j*,0_,*j*∈ [ 1,*M*] is

(12) $$ Q_{j}=\frac{\pi_{j,\,0} h_{j}}{T},  $$

where *T* is the average state holding time for all states, *h*_*j*_ is average state holding time for state *S*_*j*, 0_, and it can be calculated as

(13) $$ T=\pi_{0,\,N}+\sum_{n=0}^{N-1}{\pi_{0,\,n} \frac{1}{\lambda}}+\sum_{j=1}^{M}\sum_{i=1}^{W-1}\pi_{j,\,i}+\sum_{j=1}^{M}\pi_{j,\,0} h_{j}.  $$

When a UE triggers a random access, it resides at state *S*_*j*, 0_,*j*∈ [ 1,*M*] for 1 ms to transmit a preamble. Therefore, the probability that a UE is sending a preamble can be obtained as

(14) $$ \tau={\sum_{j=1}^{M}\frac{1}{h_{j}} Q_{j}},  $$

which eventually becomes a function of *p*_*c*_ as both *Q*_*j*_ and *h*_*j*_ are the functions of *p*_*c*_.

For a given UE, the collision probability *p*_*c*_ can be calculated as follows

(15) $$ {}p_c=\sum_{i=1}^{N_M-1}\binom{N_M-1}{i}\tau^{i}(1-\tau)^{N_M-1-i} \!\left(\!1-\left(\frac{N_C-1}{N_C}\right)^{i}\right),  $$

which is a function of *τ*.

It can be seen that formulas (14) and (15) comprise a nonlinear equation system, which could be solved by numerical methods. The pseudocode to solve this nonlinear equation system is shown in Algorithm 1. Therefore, we can compute the collision probability *p*_*c*_ and *τ*.



As mentioned above, an unsuccessful random access can be either caused by wireless channel error or by collision; hence, the expected duration for an unsuccessful random access at the *j*th attempt can be calculated as

(16) $$ T_{j}^{\prime}=\frac{p_{j}^{\prime}(1-p_{c})+p_{c}p_{E,\,j}}{p_{j}^{\prime}(1-p_{c})+p_{c}}T_{E}+\frac{p_{c}(1-p_{E,\,j})}{p_{j}^{\prime}(1-p_{c})+p_{c}}T_{C}.  $$

If a random access is successful at the first attempt, the expected channel latency *T*_1_ includes the backoff time and time to perform a successful random access. Hence

(17) $$ T_{1}=T_{S}+W/2.  $$

If a random access is successful at the *j*th try (*j*>1), the expected channel access latency *T*_*j*_ includes the latency caused by the precedent unsuccessful random access and the latency of the last successful random access. Therefore, we have

(18) $$ T_{j}=\sum_{n=1}^{j-1}[\!T_{n}^{\prime}+W/2]+T_{S}+W/2, j>1.  $$

Then, the expected time used to deliver an aggregated packet is

(19) $$ d^{\prime}=\frac{1-p_{1}}{1-\prod_{j=1}^{M}p_{j}}T_{1}+\sum_{j=2}^{M}\frac{(1-p_{j})\prod_{n=1}^{j-1}p_{n}}{1-\prod_{j=1}^{M}p_{j}}T_{j},  $$

where $p_{j}=p_{j}^{\prime }+\left (1-p_{j}^{\prime }\right)p_{c}$ is the probability of an unsuccessful random access at the *j*th try.

With the above results, the overall latency, defined as the waiting time to accumulate packets plus the time to deliver the aggregated packet, can be obtained as

(20) $$ d=\frac{N-1}{\lambda}+d^{\prime},  $$

where the $\frac {N-1}{\lambda }$ is the waiting time needed to aggregate further *N*−1 packets after receiving the first packet.

One usage of packet aggregation method is to reduce the packet loss rate. It is obvious that the preamble collision rate decreases as the number of aggregated packets increases. However, a larger aggregated packet will cause an increase in the packet transmission latency, which may not desirable for certain delay critical applications. Therefore, the optimal packet aggregation number should be dynamically adjusted so that the packet loss rate remains below the desired threshold while the latency is minimized. This optimization problem can be formulated as

(21) $$ \begin{aligned} & \underset{N}{\text{arg min}} & & d \\ & \text{subject to} && \prod_{i=1}^{M}p(i)<\alpha, \\ &&& N<N_{\text{max}}, \end{aligned}  $$

where *p*(*i*) is the packet loss rate for the *i*th transmission, *α* is the packet loss rate limit, and *N*_max_ is the maximum number of aggregated packets which is determined by the buffer size as well as power capacity^2^. As we do not have a closed form of *d*, therefore, this optimization cannot be solved by any specific optimization method. Instead, we use exhaustive search to solve this problem.

Another usage for packet aggregation is energy saving. With packet aggregation, only one random access is performed for the delivery of multiple packets, which reduces the power consumption for the transmission as well as the packet processing [16, 17]. To capture this effect for the packet transmission, here, we define an energy-saving factor *β* as

(22) $$ \beta=1-\frac{E_{t}+P_{a}T_{a}}{NE_{t}+NP_{a}T_{u}},  $$

where *E*_*t*_ is the energy used for preamble and L2/L3 message transmission in one random access, *P*_*a*_ is the average power when a UE is at a connected state (not transmitting), *T*_*a*_ is the resulted latency when packet aggregation is applied, *T*_*u*_ is the latency when the packet aggregation is not used, and *N* is the number of aggregated packets. In formula (22), *E*_*t*_+*P*_*a*_*T*_*a*_ is the power consumed for the delivery of *N* packets when packet aggregation is applied, while *NE*_*t*_+*NP*_*a*_*T*_*u*_ is power spent for the transmission of *N* packets without packet aggregation. Since a UE usually spends much more power in transmission than connected state [29], we can assume that *E*_*t*_≫*P*_*a*_*T*_*a*_ and *NE*_*t*_≫*NP*_*a*_*T*_*u*_. Therefore, we can approximate the above formula as

(23) $$ \beta=1-\frac{1}{N}.  $$

Note that when number of aggregated packets *N* goes large, *T*_*a*_ also increases; therefore, the power spent at the connected state *P*_*a*_*T*_*a*_ and *NP*_*a*_*T*_*u*_ may not be omitted.

When used for power saving, the number of aggregated packets is maximized as long as the delay constraints is satisfied. This optimization problem can be formulated as

(24) $$ \begin{aligned} & \underset{N}{\text{arg max}} & & \beta \\ & \text{subject to} && d<d_{\text{lim}}, \\ &&& N<N_{\text{max}}, \end{aligned}  $$

where *d*_lim_ is the delay limit.

### Multiple traffic sources

In the previous section, we consider the single traffic source where available random access preambles are divided into non-overlapping sets to provide per traffic source-dedicated preamble. In this section, we elaborate on the case where the available random access resources are shared among multiple traffic sources. For simplicity, we only study two traffic sources, namely realtime traffic and non-realtime traffics. The objective is to reduce the loss rate for both traffics, but with different aggregation policies allowing delay-tolerant traffic to aggregate more packets on the benefit of the delay-critical traffic. The underlying assumption is that the decision on the number of aggregated packets for both realtime and non-realtime traffic is determined by eNB and sent to each UE.

Let us denote that the number of devices with realtime traffic is *N*_*r*_ and the number of devices with non-realtime traffic is *N*_*n*_. The packet aggregation number for realtime and non-realtime devices is denoted as *a*_*r*_ and *a*_*n*_, respectively. With the proposed semi-Markov model, we can derive the probability that a device with a realtime traffic or a non-realtime traffic is sending a preamble, which are denoted as *τ*_*r*_ and *τ*_*n*_, respectively. Similarly, *τ*_*r*_ and *τ*_*n*_ are the functions of the preamble collision probability. The preamble collision probability for a realtime traffic is calculated as

(25) $$ \begin{aligned} p_{r}^{\prime}=&\sum_{i=0}^{N_r-1}\binom{N_r-1}{i}\tau_{r}^{i}(1-\tau_r)^{N_r-1-i} \\ &\times\sum_{j=0}^{N_n}\binom{N_n}{j}\tau_{n}^{j}(1-\tau_n)^{N_n-1-j} \left(\!1-\left(\frac{N_C-1}{N_C}\right)^{i+j}\right). \end{aligned}  $$

Similarly, the preamble collision probability for a non-realtime traffic can be calculated as

(26) $$ \begin{aligned} p_{n}^{\prime}=&\sum_{i=0}^{N_n-1}\binom{N_n-1}{i}\tau_{n}^{i}(1-\tau_n)^{N_n-1-i} \\ &\times\sum_{j=0}^{N_r}\binom{N_r}{j}\tau_{r}^{j}(1-\tau_r)^{N_r-1-j} \left(1-\left(\frac{N_C-1}{N_C}\right)^{i+j}\right), \end{aligned}  $$

which are functions of *τ*_*r*_ and *τ*_*n*_.

By the use of numerical calculation method, we can get the preamble collision probability $p_{r}^{\prime }$ and $p_{n}^{\prime }$ and preamble transmission probabilities *τ*_*r*_ and *τ*_*n*_. Therefore, the latency for a realtime traffic *d*_*r*_ and a non-realtime traffic *d*_*n*_, as well as the packet loss rate for a realtime *p*_*r*_ and a non-realtime traffic *p*_*n*_ can be calculated.

Table 2 shows the symbols used for multiple traffic sources.

**Table 2:** Symbols used for multiple traffic sources

Notation	Definition
*N*_*r*_	Number of devices with realtime traffic
*N*_*n*_	Number of devices with non-realtime traffic
*a*_*r*_	Packet aggregation number for real time
*a*_*n*_	Packet aggregation number for non-real time
*τ*_*r*_	The probability a device with realtime traffic is sending a preamble
*τ*_*n*_	The probability a device with non-realtime traffic is sending a preamble

As an example policy, the transmission for a non-realtime traffic can be postponed in order to reduce the latency for a realtime traffic (objective function). While the delay requirement for a non-realtime could be large, the number of aggregated packets should not be too large to violate the delay requirement (the first constraint). Moreover, similar to the single traffic source, we can constraint the packet loss rate (the second and third constraints). We can formulate our optimization problem to find the optimal packet aggregation number as

(27) $$ \begin{aligned} & \underset{a_{r}, a_{n}}{\text{arg min}} & & d_{r} \\[-2pt] & \text{subject to} && d_{n}<D_{n}, \\[-2pt] &&& p_{r}<P_{r}, \\[-2pt] &&& p_{n}<P_{n}, \\[-2pt] &&& a_{r}<A_{r}, \\[-2pt] &&& a_{n}<A_{n}, \end{aligned}  $$

where *D*_*n*_ is the delay constraint for the non-realtime traffic, and *P*_*r*_ and *P*_*n*_ are the packet loss rate threshold for the realtime and non-realtime traffics, and *A*_*r*_ and *A*_*n*_ are the maximum packet aggregation number for the realtime and non-realtime traffics. Similar to the single traffic source, packet aggregation for multiple traffic sources can also be used to save the power.

## Simulation results

To validate and evaluate the performance of the proposed method, simulations are performed with MATLAB. Both the analytical SMM model and random access procedure as specified in 3GPP 36.321 [18] have been developed for model validation and performance assessment.

### Simulation parameters

The parameters for random access are shown in Table 3. We use the value specified in [30] for contention response window *T*_RARW_ and contention resolution timer *T*_timer_ and the value specified in [18] for backoff window *W*. The value for parameter *T*_RAR_ and *T*_CR_ is obtained by assuming that the time used to decode preamble, SR message, and contention resolution message is 3 ms, respectively. Here, the number of preambles is set to 20 belonging to random access preamble subgroup A, since it is assumed that the remaining preambles are reserved or allocated to the other traffic sources [11]. We assume that random access opportunity is available in every subframe, similar to 3GPP configuration index 14 [27]. The transmission limit *M* for random access is set to 5, and the desired packet loss rate *α* is 0.1.

**Table 3:** Simulation parameters

Parameter	Description	Value
*T*_RAR_	Duration that starts at the end of a preamble’s transmission and ends at the time instant when the RAR message can be received	5 ms
*T*_D_	Time used to decode a RAR message	3 ms
*T*_RARW_	Random access response window	10 ms
*T*_timer_	Contention resolution timer	24 ms
*T*_CR_	Duration which starts at time instant when a UE sends the SR message and ends at the time instant when a UE decodes the contention resolution message	8 ms
*T*_E_	State holding time if no RAR message is received by UE (*T*_E_=*T*_RAR_+*T*_RARW_).	15 ms
*T*_C_	State holding time if a preamble is transmitted with collision and it is correctly detected by eNB (*T*_C_=*T*_RAR_+*T*_D_+*T*_timer_)	32 ms
*T*_S_	State holding time if a preamble is corrected received and without collision (*T*_S_=*T*_RAR_+*T*_D_+*T*_CR_)	16 ms
*W*	Backoff window size	30
*N*_C_	Number of preamble	20
*M*	Transmission limit	5
*α*	Required loss rate	0.1

The preamble detection rate can be obtained through physical layer simulations with specific channel model. Here we use the results from [10]: in case of no collision, the preamble detection rate is assumed to be $1-\frac {1}{e^{j}}$, where *j*∈ [ 1,*M*] indicates the *j*th preamble transmission. When a preamble are sent by multiple UEs, we assume that it can always be correctly decoded, i.e., *E*_*j*, *i*_=0 when *i*≥2. This assumption is realistic and typical in LTE/LTE-A as the probability that none of the multiple random access peaks cannot be decoded by eNB is relatively low.

### Model validation

To validate that the proposed analytical model is consistent with real random access procedure, we compare the analytical results obtained using the SMM model with the simulation results produced based on the protocol implementation. The number of aggregated packets *N* is set to 1 and 2 to represent the regular random access and random access with packet aggregation. Here, we compare preamble collision rate instead of packet loss rate. The reason is that in some cases, the packet loss rate is very small, e.g., it is very close to 0 when *λ*=1/300,*N*=2, and the number of UE is less than 1500. Therefore, the difference between simulated results and analytical results might not be clearly seen when comparing.

**Fig. 3:**
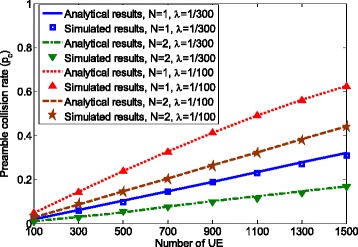
Model validation in terms of preamble collision rate

**Fig. 4:**
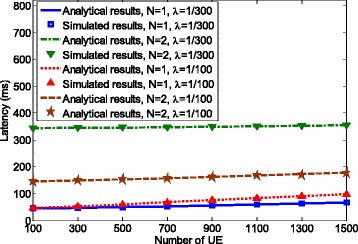
Model validation in terms of latency

Figures 3 and 4 show the preamble collision rate and latency for packet arrival rate *λ*=1/100 and *λ*=1/300 (packet/ms). It can be seen that the analytical results match the simulation results, which validates the proposed SMM model.

### Packet aggregation for single traffic source

To evaluate the performance of the packet aggregation for the case of single traffic, we vary the number of UEs and the packet arrival rate and analyze the number of aggregated packets, packet loss rate, latency, and power saving factor. In this case, the threshold for the packet loss rate *α* is set as 0.1, and the maximum number of aggregated packets *N*_max_ is 50. The average packet interval is 100, 300, and 500 ms, which is much larger than the backoff window size.

**Fig. 5:**
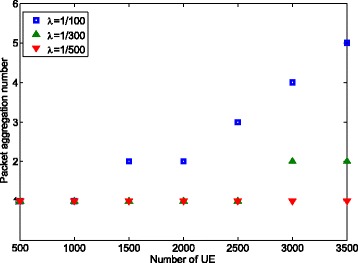
Number of aggregated packets for single traffic case

Figure 5 shows the amount of aggregated packets under different number of UEs and packet arrival rate *λ* (packets/ms). It can be seen that the amount of aggregated packets is *non-decreasing* with the increase of packet arrival rate or number of UE. This *adaptive* behavior is desirable since the collision rate increases with packet arrival rate or number of UE. Thus, if the collision rate becomes larger than the threshold, more packets will be aggregated to lower the collision. Otherwise, the amount of aggregated packets remains the same. As shown in Fig. 5, when *λ* = 1/100 and the number of UE is 2000, the amount of aggregated packets is 2 and the packet drop rate is 0.093 which is very close to the threshold 0.1. Therefore, when the number of UE increases to 2500, the amount of aggregated packets increases to 3, which reduces the packet drop rate to 0.07. We also notice that the packet aggregation number is always 1 when *λ*=1/500. The reason is that the packet loss rate is always less than the threshold (0.1) as the number of UEs increases. For example, the loss rate is 0.05 when *λ*=1/500 and number of UE is 3500.

We observe that the packet aggregation number is different even if the average number of new transmission is the same. For example, the packet aggregation number is 1, when *λ*=1/100, and the number of UE is 1000 (the average number of new transmissions in one subframe is 10). In contrast, the packet aggregation number is 2, when *λ*=1/300, and the number of UE is 3000 (the average number of new transmissions in one subframe is also 10). This is because not all the newly generated packets will trigger a new random access attempt, but rather, they may be aggregated and delivered with the same random access process. To illustrate further using the example above, we notice that the latency is 80 ms when *λ*=1/100 and number of UE is 1000. Hence, 55 % of the packets are generated during the random access attempts (1−*e*^(−80/100)^=0.5507) and will be delivered using the ongoing transmission. As a result, only 45 % of the newly generated packet will trigger random access attempts. For the other case, the latency is 100 ms when *λ*=1/300 and number of UE is 3000, making the percentage of packets generated during an ongoing random access smaller, i.e., 28 % (1−*e*^(−100/300)^=0.28). Subsequently, 72 % of the newly generated packets will trigger a new random access attempt, which is much higher than the former case. As a result, the packet aggregation number is set to 2 to lower the packet loss rate.

**Fig. 6:**
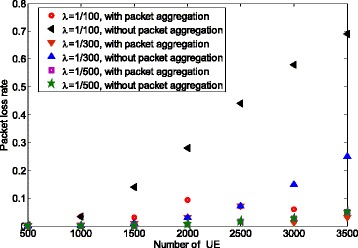
Packet loss rate for single traffic case

Figure 6 shows the packet loss rate corresponding to the packet aggregation results shown in Fig. 5. It can be observed that the packet loss rate always remains lower than the threshold (0.1), which validates the method. In contrast, without packet aggregation, the packet loss rate becomes very high and exceeds the maximum packet loss rate in particular when *λ*=1/100 or when *λ*=1/300 and the number of UE is larger than 2000.

**Fig. 7:**
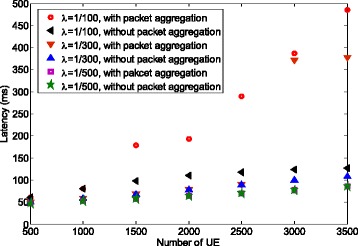
Latency for single traffic case

As discussed above, we lower the packet loss rate at the expense of latency increase. Figure 7 compares the channel access latency with or without packet aggregation. We can see that the latency is increased when using packet aggregation. For example, when comparing for 2000 UEs and *λ*=1/100, the latency is increased from 110 ms for no aggregation to 193 ms with aggregation level 2. We can conclude that if the resulted latency is larger than delay constraint with the packet aggregation, more preambles and/or physical random access channel (PRACH) resources should be allocated by eNB. This is because with more preambles and/or preamble resources, the preamble collision rate is reduced. Therefore, a UE aggregates less packet and hence the delay constraint may be satisfied. For non-realtime applications with elastic delay constraint, packet aggregation method can significantly reduce the packet loss rate to a very small value.

### Power saving using packet aggregation for single traffic source

In this part, we show the power saving ratio that can be obtained by the usage of the packet aggregation method using the formula (24). Here, we set the delay limit to 300 ms, which is related to one of the delay requirements for non-guaranteed bit rate (GBR) bears in LTE/LTE-A [19]^3^. Since the delay requirement is 300 ms, packet aggregation is not applicable for the traffic where *λ* = 1/300 (packet/ms) or 1/500. Therefore, we only consider the case where *λ* = 1/100.

**Fig. 8:**
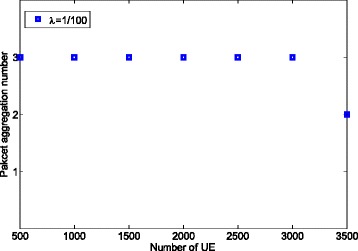
Amount of aggregated packets used for power saving

Figure 8 shows the number of aggregated packets as a function of the number of UEs. It can be observed that the packet aggregation level is changing from 3 to 2 when the number of UE becomes greater than 3000, which in turn causes the power saving factor to drop from 0.67 to 0.5. Note that in this case, the objective is to *maximize* the number of aggregated packets under the delay constraint, which is different from the previous case where one of the constraints is to limit the loss rate (see Fig. 5).

**Fig. 9:**
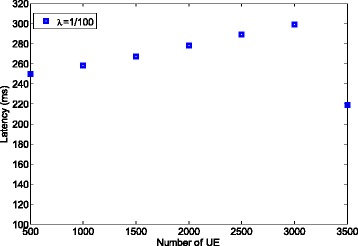
Latency induced for power saving

Figure 9 demonstrates the induced latency based on the results shown in Fig. 8. It can be observed that the delay is constantly less than the limit (300 ms). We also notice that when the number of UE is 3000, the resulted latency is very close to 300 ms. Consequently, when the number of UE further increases to 3500, the packet aggregation number is decreased to 2 in order to maintain the delay constraint.

**Fig. 10:**
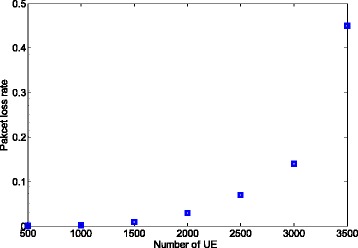
Packet loss rate induced for power saving

Figure 10 shows the packet loss rate when using the results shown in Fig. 8. We find that the packet loss rate is less than 0.1 when the number of UE is no larger than 2500. However, it increases to 0.14 when the number of UE is 3000 and it is 0.45 when the number of UE is 3500. Assuming that the packet loss requirement is 0.1, the packet loss requirement is *violated* while the delay requirement is maintained. To address the problem where the delay requirement and packet loss requirement cannot be satisfied at the same time, more preambles and/or PRACH resources should be allocated to reduce the collision rate and subsequently the packet loss. Hence, a UE does not need to aggregate a large number of packets. As a result, the delay constraint can be satisfied.

### Packet aggregation for multiple traffic sources

In this section, we consider the case where the available random access resources are shared among multiple traffic sources. For simplicity, we only study two distinct traffics, namely realtime traffic and non-realtime traffics, with the objective of reducing the loss rate for both traffics using different aggregation policies.

To highlight the packet aggregation trade-off between realtime and non-realtime traffics, we apply the packet arrival rate of 1/100 for realtime traffic and 1/300 for non-realtime traffic. Note that for a larger packet arrival rate, e.g., 1/500, the trade-off occurs for a larger number of UEs. We assume that the delay requirement for non-realtime traffic is 5000 ms and that the number of UEs which transmit non-realtime traffic is set to 10,000. The number of UE which sends realtime traffic varies from 500 to 1500. The delay limit for realtime traffic is not specified. Instead, we want to minimize the latency for realtime traffic as shown in formula (27).

The packet loss rate for realtime and non-realtime traffic is set to 0.1, and the maximum number of aggregated packets for realtime and non-realtime traffics is set to 20 and 50, respectively.

**Fig. 11:**
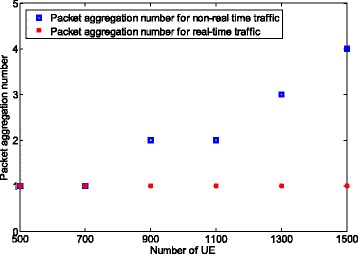
Number of aggregated packets for realtime and non-realtime traffic for multiple traffic types

Figure 11 presents the number of aggregated packets with realtime and non-realtime traffic. We observe that the packet aggregation number for UEs with non-realtime traffic increases when the number of UEs with realtime traffic changes from 500 to 1500. The reason is that the packet loss rate increases with the number of UE, which requires higher aggregation level to reduce packet loss rate. Moreover, we also find that no packet aggregation is performed for the realtime traffic (i.e., packet aggregation number remains 1). The reason behind this behavior relies on the objective function in formula (27), which minimizes the latency for realtime traffic. However, it has to be noted that the packet aggregation number for realtime traffic is also subject to increase when the number of UEs increases. The reason is that when the delay for the non-realtime traffic reaches the limit, UEs with realtime traffic will also start to aggregate packet to reduce the overall collision.

**Fig. 12:**
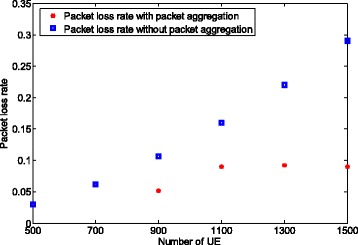
Packet loss rate comparison with and without packet aggregation for multiple traffic types

Figure 12 shows the packet loss rate when using the packet aggregation results presented in Fig. 11. For comparison, we also plot the packet loss rate without packet aggregation. We find that without the packet aggregation method, the packet loss rate increases with the number of UEs with realtime traffic. We notice that the packet loss rate increases from 0.04 to 0.29 as the number of UE increases, which violates the loss rate constraint. To comply with packet loss requirement, the packet aggregation policy joins four packets for the UE with non-realtime to keep the packet loss rate below the desired threshold (see Fig. 11).

**Fig. 13:**
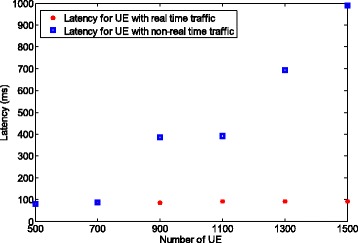
Latency comparison for realtime and non-realtime traffic for multiple traffic types

Figure 13 shows the latency when using the packet aggregation results presented in Fig. 11. The latency for the UE with non-realtime traffic increases greatly from 79 to 991 ms when the number of UE increases from 500 to 1500. This phenomenon is the consequence of the aggregation policy instructed by the eNB allowing UEs with non-realtime traffic to aggregate more packets as the number of UE increases, which in turn increases the latency. In contrast, the latency for the UEs with realtime traffic remains almost constant (less than 100 ms) with the increase in the number of UEs. This is a desirable property for the realtime applications. It can be concluded that if the delay limit for non-realtime traffic becomes very large, the delay for realtime traffic can mostly be kept as small as possible.

## Discussion

In this paper, we propose an adaptive packet aggregation method to reduce packet loss rate and/or power consumption at the expense of an extra delay. Through extensive simulations, we find that:

- For delay-tolerant traffics, packet loss rate and/or transmit power can be minimized until the maximum aggregation number is reached, which should be less than the memory limit.
- For delay-critical traffics, packet loss rate and/or transmit power can be minimized until the delay threshold is reached.
- For multiple traffic sources, the packet aggregation levels have to be jointly optimized to minimize packet loss rate and/or transmit power for delay-tolerant and delay-critical traffic sources.

Results also reveal that although the packet aggregation can improve the channel access efficiency, an aggregated packet becomes more sensitive to bursty wireless channel errors as the packet size increases. In addition, memory requirement to store the aggregated packets may increase the cost of end devices and depending on the device category, this may not always be available (in particular when the maximum number of aggregated packets is large or when packets are large).

Finally, the packet aggregation method can be easily applied to LTE/LTE-A with minimal modifications (no modification to PHY/MAC layers). It is an efficient yet simple technique that can be used to improve the performance for the current and future cellular systems along with the following directions. First, when there are massive number of devices in a cell, the preamble collision rate becomes high, and as a result, the reliability of random access becomes lower (the network becomes congested [31]). With packet aggregation, the collision rate can be greatly reduced and the network congestion can be avoided. Second, it reduces the number of random access attempts in the end devices, which in turn saves power. Third, the packet aggregation method is highly applicable when the packet size is very small, e.g., less than 30 bytes, in which case the signaling overhead to schedule the packet becomes dominant with respect to the payload. Moreover, the minimum resource block in LTE/LTE-A contains 12 subcarriers with a duration of 1 ms (14 OFDM symbols for normal cyclic prefix length), which might be too large for transmission of small-sized packet. By the use of packet aggregation, packet size becomes larger, which alleviates the signaling overhead.

## Conclusions

To limit the packet loss rate and improve the reliability for random access, we introduce an adaptive packet aggregation method that allows a device to start a random access process only when the number of aggregated packets reaches the given threshold. This method introduces an extra channel access latency, which is used to accumulate a certain amount of packets. In order to find the optimal packet aggregation number which minimizes the packet loss rate while maintaining the delay requirement, we employ a semi-Markov process model to analyze the random access procedure with packet aggregation. Moreover, we extend the packet aggregation method to increase the energy efficiency for battery-powered devices. This allows a device to reduce the number of random access attempts by aggregating multiple packet deliveries into a single transmission, which in turn saves the power.

We carried out extensive simulations to validate the proposed semi-Markov process model and to evaluate the performance of the proposed method. Through simulations, we find that (i) the analytical results obtained using the semi-Markov chain model match with the simulation results produced based on the protocol implementation, (ii) the amount of aggregated packets is optimally selected and adapted to number of devices, traffic load, and delay constraints, and (iii) the packet loss rate and/or power consumption are greatly reduced.

## Endnotes

^1^ Throughout this paper, the terms device and UE are used interchangeably.

^2^ A UE saves more power when *N* becomes larger. Therefore, for power-limited MTC device, *N*_max_ tends to be large.

^3^ The packet delay limit specified for non-GBR bear includes the delay of air interface as well as of core network. Since the delay of air interface is usually much larger than that of core network, we use this value as the delay for air interface for approximation.
